# The Campaign of the London Medical Committee

**Published:** 1913-01-25

**Authors:** 


### NATIONAL INSURANCE ACT DEPARTMENT.

### XL.—The Campaign of the London Medical Committee.

We understand that, in spite of the decision of the representative meeting of the British Medical Association releasing those members who were pledged against panel service, the London Medical Committee intends to continue its campaign against service under the Act. The principal contention of this Committee is that every insured person has the right of free choice of doctor, and that this should be insisted upon, even though the doctor chosen has not joined the panel. During the past few days placards have appeared on the hoardings calling upon insured persons to insist on this freedom of choice.

The panel system was the method contemplated by the Act for the administration of medical benefit, but provisions were inserted to meet the case of a possible failure of that system, and also to satisfy the special circumstances of insured persons to whom the panel system, when adopted, might not apply. Now the panel system has been adopted, and under it any duly qualified medical practitioner has the right of having his name included in the list. Furthermore, every insured person entitled to medical benefit has the right of selecting from the list appropriate to his district the doctor by whom he wishes to be attended and treated. This choice is limited to the doctors whose names are on the panel, and is subject to the doctor consenting to treat him. Of course, where the practitioners have formed an adequate panel, it is seen that freedom of choice is amply secured. In some cases, however, particularly when the majority of the doctors in a given district have not joined the panel, the choice must be seriously curtailed. To meet these circumstances it is possible for the Insurance Committees to allow insured persons to make their own arrangements for receiving medical treatment.

The provision of the Act relating to the matter is contained in Section 15, sub-section (3), which is of a complicated character. It is laid down that the regulations made by the Insurance Commissioners for the administration of medical benefit must confer on the Insurance Committees the power to allow any insured persons to make their own arrangements for receiving medical attendance and treatment, including medicines and appliances, instead of receiving these benefits under the panel system. A point that must here be noted is that the authority thus delegated to the Insurance Committees is permissive only, and apparently the Committees will be entitled to treat such applications as they may receive under the sub-section exactly as they choose. When the Committee agrees to allow insured persons to make their own arrangements it will have to contribute out of its medical benefit funds towards the cost of treatment of such persons, but the sums so expended must not exceed in the aggregate the amount that would have been paid if they had not made their own arrangements. It is thus clear that no financial advantage can accrue to the doctors in the aggregate by abstaining from joining the panels, except in so far as they are able to obtain additional payments of a private nature for services rendered from those persons who have made their own arrangements.

It must further be remembered that in the councils of the Insurance Committees the representatives of the approved societies have the controlling voice, and it has already been broadly hinted that the permission to make arrangements with doctors off the panel will only be granted in exceptional cases.

One word remains to be said to insured persons. The doctors serving on the panels are to be remunerated according to the number of insured persons on their books, whether they are well or ill. The pink ticket that has now been furnished to insured persons entitling them to claim medical benefit has to be signed by the doctor whom they choose, and his signature should be obtained at once. It is not necessary to wait until illness occurs. If the matter be deferred until treatment is required it may happen that as no choice has been made your name will have been put on the list of a doctor of whom you know nothing. A little trouble may be caused in obtaining the doctor's signature to the ticket, but the trouble will well be repaid to yourself in the satisfaction you will obtain, and the doctor will not lose the remuneration you intended.

